# Influence of the Sample Preparation Method in Discriminating *Candida* spp. Using ATR-FTIR Spectroscopy

**DOI:** 10.3390/molecules25071551

**Published:** 2020-03-28

**Authors:** Savithri Pebotuwa, Kamila Kochan, Anton Peleg, Bayden R. Wood, Philip Heraud

**Affiliations:** 1 Department of Microbiology, Monash University, Clayton, Victoria 3800, Australia; savi.pebotuwa@monash.edu; 2 Centre for Biospectroscopy and School of Chemistry, Monash University, Clayton, Victoria 3800, Australia; Bayden.Wood@monash.edu; 3 Infection and Immunity Program, Monash Biomedicine Discovery Institute and Department of Microbiology, Monash University, Clayton, Victoria 3800, Australia; anton.peleg@monash.edu; 4 Department of Infectious Diseases, The Alfred Hospital and Central Clinical School, Monash University, Melbourne, 3004, Victoria 3800, Australia; 5 Department of Microbiology and the Biomedicine Discovery Institute, Monash University, Clayton, Victoria 3800, Australia

**Keywords:** ATR-FTIR spectroscopy, fungi, PCA, *Candida*

## Abstract

Several studies have investigated the capacity of ATR-FTIR spectroscopy for fungal species discrimination. However, preparation methods vary among studies. This study aims to ascertain the effect of sample preparation on the discriminatory capacity of ATR-FTIR spectroscopy. *Candida* species were streaked to obtain colonies and spectra were collected from each preparation type, which included: (a) untreated colonies being directly transferred to the ATR crystal, (b) following washing and (c) following 24-h fixation in formalin. Spectra were pre-processed and principal component analysis (PCA) and K-means cluster analysis (KMC) were performed. Results showed that there was a clear discrimination between preparation types. Groups of spectra from untreated and washed isolates clustered separately due to intense protein, DNA and polysaccharide bands, whilst fixed spectra clustered separately due to intense polysaccharide bands. This signified that sample preparation had influenced the chemical composition of samples. Nevertheless, across preparation types, significant species discrimination was observed, and the polysaccharide (1200–900 cm^−1^) region was a common critical marker for species discrimination. However, different discriminatory marker bands were observed across preparation methods. Thus, sample preparation appears to influence the chemical composition of *Candida* samples; however, does not seem to significantly impact the species discrimination potential for ATR-FTIR spectroscopy.

## 1. Introduction

There has been a significant increase in incidence and prevalence of fungal infections in humans since the 1980s [1]. Of the fungi infecting humans, those of the genus *Candida* are most common and present clinically as superficial or systemic infections [2]. *Candida* species are commonly found as commensals in the gastrointestinal tract, oral cavity, esophageal tract, skin and genitourinary tract [1]. The rise in incidence of Candidiasis can be attributed to the increased number of immunocompromised patients, invasive procedures and inappropriate use of antibiotics [3].

*Candida albicans* is the most commonly isolated species from all forms of Candidiasis. However, recently there has been a mycological shift to non-*albicans Candida* species (NAC) such as *Candida glabrata, Candida parapsilosis* and *Candida krusei* [4,5,6,7].

*Candida* species differ in their antifungal susceptibility and virulence profiles, as such, diagnosing the infecting species is important for clinical management [3,8]. The current gold standard for candidiasis diagnosis is blood culture (BC). This takes up to 24–48 h to show a positive result, and discriminating down to species level using differential media such as CHROMagar delays treatment even further [9,10,11]. Moreover, studies have shown that a delay in administering appropriate therapy by 12–48 h is associated with significant increase in mortality [12,13,14,15]. Hence, an efficient method for *Candida* species identification would be a major advance for patient treatment.

Vibrational spectroscopy is a non-destructive tool used for the elucidation of vibrational energy in a substance. There are two basic types of vibrational spectroscopy, these include infrared and Raman spectroscopy. Several studies have explored the capacity of infrared spectroscopy, in particular Fourier transform infrared spectroscopy (FTIR) for bacterial typing; however, only a handful of studies have been conducted with respect to their discriminatory capacity for *Candida* species [16,17,18,19,20,21,22,23,24,25]. Furthermore, amongst these, there is a significant variability in sample preparation of *Candida* species for spectroscopic measurements. These include directly transferring untreated colonies from agar plates to an ATR crystal (or zinc selenide window) or suspending them in distilled water prior to placement on the crystal [19,22,23].

Consequently, this study aims to investigate the influence of different preparation methods on the capacity of ATR-FTIR spectroscopy for discriminating *Candida* species and whether discriminatory markers vary for each preparation method. For this purpose, three different preparation methods have been investigated. Principal component analysis (PCA) was performed to ascertain the effect of preparation method on spectral profile, and afterwards, the capability of each preparation method in discriminating between four species of *Candida* was investigated. K-means cluster analysis (KMC analysis) was then performed to visualise the relationship of spectra in each preparation method.

## 2. Results

### 2.1. Effects of Preparation Method on Species Discrimination

**Figure 1:**
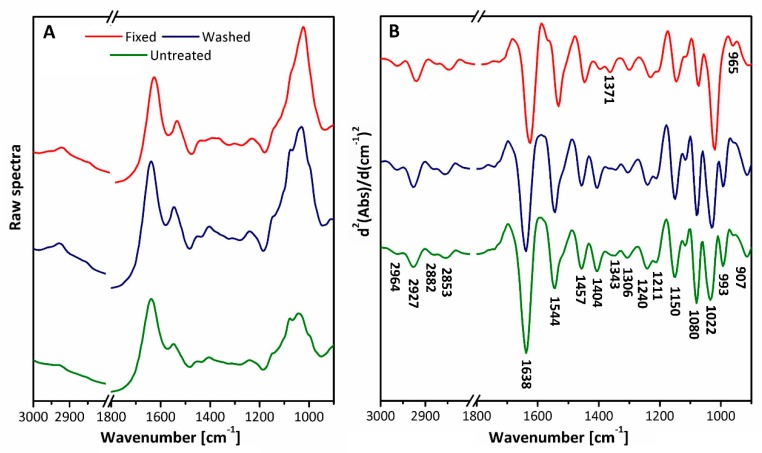
Attenuated total reflectance Fourier transform infrared (ATR-FTIR) spectra for *Candida glabrata* for fixed, untreated and washed samples that are: (**A**) raw data; one technical replicate per preparation method is shown; (**B**) mean pre-processed data for each preparation method, significant bands are labelled.

Figure 1 includes the raw and pre-processed spectra of *Candida glabrata* for the different preparation methods. Significant bands are shown in the pre-processed data, and the corresponding assignments are outlined in Table 1.

The most significant spectral differences observed between preparation types in the average second derivative spectra occurs with bands at 1404 cm^−1^, assigned to the COO- symmetric stretching (ν_s_ COO-) from carboxylic acids and free amino acids of lipids and proteins; at 1371 cm^−1^, assigned to the CH_2_ wagging in lipids and β-1,3 glucans of lipids and polysaccharides; at 1343 cm^−1^, assigned to CH_2_ wagging vibrations of lipids; at 1022 cm^−1^, assigned to β-1,4 glucans of polysaccharides; at 993 cm^−1^, assigned to β-1,6 glucans of polysaccharides; and at 965 cm^−1^, assigned to mannans and the C–O stretch (νC–O) of phosphodiesters and ribose of DNA and polysaccharides.

**Figure 2:**
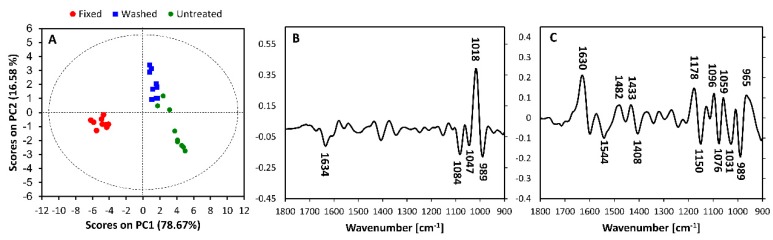
Principal component analysis (PCA) of Principal Components (PC) PC1 versus PC2 with (**A**) scores plot and loadings plot for (**B**) PC1 and (**C**) PC2 for spectra from *Candida glabrata* subjected to the three different preparation methods. PCA and PC1 loadings plots showing the sample preparation dependent discrimination for all *Candida* spp. used in this study are presented in Supplementary Materials (S1).

Following this, PCA was performed on spectra from *Candida glabrata* species with the three preparation methods. Figure 2 depicts the PCA scores plot and loadings plot corresponding to PC1 versus PC2. The PCA and PC1 loadings plot of all preparation types with all species is shown in Supplementary Materials (S1).

PCA is an unsupervised multivariate data analysis (MVDA) method that is exploratory in nature, which means that it is used to differentiate between data without a priori knowledge. These techniques are useful in sorting data and identifying the differences, heterogeneity and complexity of unknown datasets [31,32]. For PCA data analysis, scores plots are interpreted in conjunction with their corresponding loadings plots. Loadings plots display variables or spectral features that contribute to most of the clustering patterns observed in the principal components (PC) of the scores plots. As spectra were converted to their second derivatives, samples that are positively scored in the scores plot are correlated with negatively loaded bands in the corresponding PC loadings plot. Likewise, negatively scored samples in the scores plot correlate with positively loaded bands in the corresponding PC loadings plot.

Investigation of the PC1 loadings plot revealed clear discrimination between sample preparation methods. Across the PC1 versus PC2 scores plot, the untreated and washed isolates clustered together along PC1 due to loadings bands at 1634 cm^−1^, 1084 cm^−1^, 1047 cm^−1^ and 989 cm^−1^, which were assigned to proteins, DNA and polysaccharides, whilst fixed isolates clustered separately due to an intense loadings band at 1018 cm^−1^, which was assigned to polysaccharides.

The untreated and fixed isolates clustered together along PC2 due to loadings bands at 1630 cm^−1,^ 1482 cm^−1^, 1433 cm^−1^, 1178 cm^−1^, 1096 cm^−1^, 1059 cm^−1^ and 965 cm^−1^, which were assigned to proteins, lipids and proteins, DNA and polysaccharides. Washed isolates clustered due to intense loading bands at 1544 cm^−1^, 1408 cm^−1^, 1150 cm^−1^, 1076 cm^−1^, 1031 cm^−1^ and 989 cm^−1^, which were assigned to proteins, lipids and proteins and polysaccharides and DNA.

### 2.2. Principal Component Analysis for Species Discrimination

**Figure 3:**
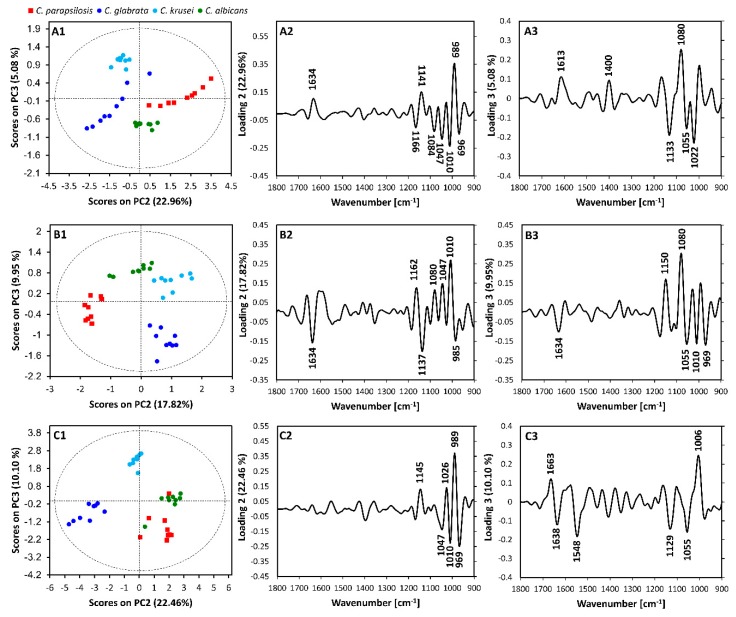
Principal Components (PC) PC2 versus PC3 scores plot for each preparation type: (**A1**) untreated (**B1**) fixed and (**C1**) washed and their corresponding loadings for PC2 (**A2**,**B2**,**C2**) and PC3 (**A3**,**B3**,**C3**).

PCA was performed using spectra from each preparation method to investigate the capacity for species discrimination, as illustrated in Figure 3. In some instances, the differences between spectra were relatively subtle and will not appear in the PC depicting the most variation (PC1). In datasets from all preparation methods PC2 showed the best discrimination between clusters of spectra assigned to different *Candida* species. PC1 seemed to show differences between species replicates and PCA scores plots, and the corresponding PC1 loadings are shown in Supplementary Materials (S2).

Investigation of the PCA scores plots of PC2 versus PC3 (Figure 3) revealed that each preparation method demonstrated similar species discrimination. *Candida krusei* and *Candida glabrata* clustered together whilst *Candida parapsilosis* and *Candida albicans* also clustered together. However, different marker bands contributed to the discrimination seen in each preparation type (Table 2).

Nonetheless, across all preparation types, loadings plots revealed that bands 1141 cm^−1^ of untreated isolates, 1137 cm^−1^ of fixed isolates and 1145 cm^−1^ of washed isolates, which corresponded to the C–O stretching of carbohydrates, contributed to the separation of clusters of *Candida krusei* and *Candida glabrata* spectra in scores plots. Additionally, bands 989 cm^−1^ of the untreated and washed isolates and band 985 cm^−1^ of fixed isolates, which corresponded to β-1,6 glucans, also contributed to the separation of clusters in the spectra of these species.

Similarly, bands 1047 cm^−1^ and 1010 cm^−1^, which corresponded to mannans and the C–O stretching of carbohydrates, respectively, contributed to the separate clustering of *Candida albicans* and *Candida parapsilosis* spectra.

### 2.3. KMC Analysis

**Figure 4:**
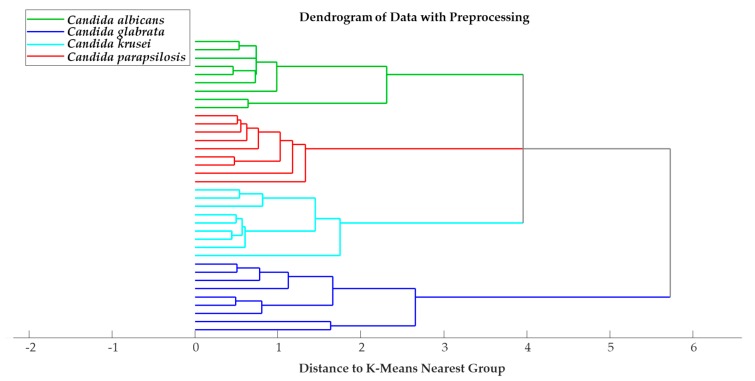
K-means cluster analysis plot for untreated dataset.

To visualise clustering patterns, KMC analysis was conducted. This technique is also an unsupervised MVDA method and is useful for qualitative analysis. It aids in observing the relationships between spectra rather than investigating the spectral features that contribute to separation. KMC analysis was performed for each preparation in spectral windows in the ranges of 3000–2800 cm^−1^ and 1800–900 cm^−1^. The best separation was achieved using the polysaccharide region, 1400–900 cm^−1^. The untreated isolates provided the best classification, as illustrated in Figure 4, with each species forming individual clusters. K-means cluster analysis plots for the washed and fixed datasets are presented in Supplementary Materials (S3).

## 3. Discussion

In this study, the majority of 4 *Candida* spp. were well discriminated using ATR-FTIR spectroscopy with three different preparation methods. There also appeared to be a clear discrimination between preparation methods; however, within each sample preparation type, similar species discrimination was achieved. The polysaccharide region was the common critical discriminatory spectral region; however, each preparation method also displayed unique marker bands. To the best of our knowledge, no previous studies have attempted to investigate the effect of sample preparation on *Candida* spp. discrimination using ATR-FTIR spectroscopy.

There seemed to be a clear separation of preparation types signifying that sample preparation had influenced the macromolecular composition of *Candida* samples. In PC1, the untreated and washed samples showed intense protein, DNA and polysaccharide bands, whilst the fixed samples showed intense polysaccharide bands. In PC2 the untreated and fixed samples showed intense protein, lipid and protein and DNA and polysaccharide bands, whilst the washed isolates showed intense protein, lipid and protein and polysaccharide and DNA bands. This could be due to the washing step damaging the integrity of cell wall polysaccharides or the loss of some water-soluble polysaccharides. Fixation may also affect the integrity of proteins.

The discrimination observed in each preparation type is consistent with previous literature. Silva et al. [19] demonstrated that *Candida albicans* and *Candida parapsilosis* clustered together and *Candida glabrata* and *Candida krusei* clustered together in PCA scores plots. These findings are also consistent with phylogenetic relationships between the four species based on a study conducted by Diezmann et al. [33], which, in a combined maximum analysis, places *Candida albicans* and *Candida parapsilosis* in the same clade and *Candida glabrata* and *Candida krusei* in a separate one, based on six genes (ACT1,EF2,RPB1,RPB2,18S rDNA and 26S rDNA) [19].

There appears to be a consensus among existing literature that the best interspecies discrimination is achieved in the polysaccharide region, thereby highlighting that polysaccharide profiles are species specific [19,22]. Findings from this study are consistent with this. Across preparation types, bands corresponding to β-1,6 glucans (985 cm^−1^, 989 cm^−1^), mannans (1047 cm^−1^) and the C–O stretching of carbohydrates (1010 cm^−1^, 1141 cm^−1^, 1137 cm^−1^ 1145 cm^−1^) contributed to the observed discrimination. Glucans and mannans are components of the fungal cell wall and these findings highlight their role in discrimination.

Each preparation type enabled species discrimination. Across preparation types, bands corresponding to C–O stretch of carbohydrates and β-1,6 glucans contributed to the clustering of *Candida krusei* and *Candida glabrata*, whilst C–O stretching of carbohydrates and mannans contributed to the clustering of *Candida parapsilosis* and *Candida albicans*. This highlights that these bands contribute most to species discrimination, regardless of preparation type.

Furthermore, for the untreated and fixed isolates, the amide I band at 1634 cm^−1^, attributed to β-pleated sheet components, was observed for the clustering of *Candida glabrata* and *Candida krusei*, whilst the C–O stretching band at 1166/1162 cm^−1^ and a band corresponding to the phosphodiester stretch at 1080/1084 cm^−1^ was observed in the clustering of *Candida albicans* and *Candida* *parapsilosis*. The band corresponding to mannans and the C–O stretch of phosphodiesters and ribose was also observed at 969 cm^−1^ in the untreated and washed isolates for these species. Similarly, the band corresponding to β-1,4 glucans at 1026 cm^−1^ was observed in the washed *Candida krusei* and *Candida glabrata* isolates but was not observed in the corresponding fixed and untreated datasets. This highlights the fact that although each preparation method provides similar species discrimination, the marker bands are unique to each preparation method.

Here we demonstrated the influence of sample preparation on the spectral profile of *Candida*, with clear-cut discrimination between different preparation methods. Despite this, within each sample preparation, species discrimination was achieved, predominantly based on the polysaccharide region. We identified several discriminatory bands, universal to sample preparation methods (1145 cm^−1^, 1141 cm^−1^, 1137 cm^−1^, 1047 cm^−1^, 1010 cm^−1^, 989 cm^−1^ and 985 cm^−1^), along with several discriminatory bands specific to selected sample preparation methodology. This highlights the need for standardisation of a sample preparation strategy, particularly in the context of future potential clinical application.

## 4. Materials and Methods

### 4.1. Fungal Collection

Four *Candida* species were obtained from a repository at the Monash Department of Microbiology and used for species discrimination. These isolates were stored in 25% glycerol broth at −80 °C and streaked to obtain colonies for spectral collection. Table 3 shows the strain information for each of the *Candida* species used for this study.

### 4.2. Culture Conditions

*Candida* species were cultured on standard yeast extract peptone dextrose (YPD) plates that were prepared in the Monash Department of Microbiology. YPD was selected as it is a complete medium for yeast growth and is most commonly used for growing yeast under non-selective conditions. All stock plates were incubated at 30 °C for 24 h.

### 4.3. Sample Preparation

Three sample preparation strategies were used: (1) direct transfer of untreated colonies for YPD onto the ATR crystal using a sterile loop; (2) transfer of colonies from YPD plate to an Eppendorf tube, followed by washing with 500 µL of ultrapure Milli-Q water and centrifugation (3000 g × 5 min.) conducted in triplicate; and (3) transfer of colonies from YPD plate to an Eppendorf tube followed by 24 h fixation in 1 mL of 10% neutral buffered formalin followed by washing in ultrapure Milli-Q water (as described in (2)). For each preparation type, 3 biological replicates were prepared, grown on separate YPD plates.

### 4.4. ATR-FTIR Spectroscopy

Samples from each respective preparation method were placed on an ATR crystal and dried using the cool setting of a blow-dryer. As yeast cells are generally around 4–6 × 6–10 µm (*Candida albicans*), sample thickness was estimated to be a minimum of 6 µm [3]. Spectra were acquired using a Bruker Alpha FTIR instrument (Bruker Optics, USA) with a diamond crystal attenuated total reflectance (ATR) accessory. An ATR correction was applied to all data.

For each prepared sample, 3 technical replicates were collected. Spectra were acquired over the wavenumber interval 4000 cm^−1^ to 600 cm^−1^ at a spectral resolution of 8 cm^−1^. For each spectrum, 64 interferograms were co-added. Prior to acquisition of each new spectra, a spectrum of the background, from a cleaned ATR crystal, was obtained to account for experimental conditions (such as changes in atmospheric CO_2_ and H_2_O). For each background spectrum, 128 interferograms were co-added.

### 4.5. Data Pre-Processing

Prior to analysis, all spectra were pre-processed by conversion into their second derivatives using the Savitzky-Golay method, smoothed using 11 smoothing points and then normalized using the standard normal variate (SNV). Mean spectra for each preparation type and each species with each preparation type was generated for further analysis.

### 4.6. PCA and KMC Analysis

Following pre-processing, data were mean-centred and analysed with PCA using MATLAB 9.1 release 2016b (MathWorks, Natick, MA, USA), using functions in the proprietary PLS_Toolbox package (Eigenvector Research Inc., Manson, WA, USA). Several spectral regions were tested for analysis: 3000–2800 cm^−1^, 1800–900 cm^−1^ and 1400–900 cm^−1^. Outliers with high leverage on the influence plot were excluded (this constituted 0.03% of the dataset) and different combinations of PCs were tested (for example, PC1 vs. PC2, PC2 vs. PC3). For this analysis, 3 PCs were chosen. The vector combination producing the most robust separation of classes was subsequently chosen for further analysis. KMC analysis was also performed on the pre-processed data in different spectral regions for each preparation type.

## 5. Conclusions

This study shows that the preparation methods of *Candida* spp. for ATR-FTIR spectroscopy significantly affects the spectral profile; however, this does not alter the species discrimination capacity of ATR-FTIR spectroscopy. PCA analysis shows that discrimination was achieved in each preparation type mainly in the polysaccharide regions, presumably due to species-specific cell wall structures. However, although each preparation type demonstrated a similar discrimination, the bands that contributed to discrimination varied for each preparation type. These findings outline how these preparation methods do not seem to affect drastically the discrimination of these *Candida* spp. for ATR-FTIR spectroscopy.

## Figures and Tables

**Table 1:** Main absorption bands and assignments for ATR-FTIR spectra of *Candida glabrata* [26,27,28,29,30].

Band cm^−1^	Functional Group	Macromolecules
2964	CH_3_ asymmetric stretch (ν_as_CH_3_)	Proteins
2927	CH_2_ asymmetric stretch (ν_as_CH_2_)	Lipids
2882	CH_3_ symmetrical stretch (ν_s_CH_3_)	Proteins
2853	CH_2_ symmetric stretch (ν_s_CH_2_)	Lipids
1741	C=O stretching vibrations (νC=O) of lipid ester carbonyl	Lipids
1638	Amide I	Proteins
1544	Amide II	Proteins
1515	Tyrosine	Proteins
1457	CH_3_ asymmetrical deformation of cellular proteins (δ_as_ CH_3_)	Proteins and lipids
1404	COO- symmetric stretching (ν_s_COO-) from carboxylic acids and free amino acids	Lipids and proteins
1371	CH_2_ wagging in lipids and β-1,3 glucans	Lipids and Polysaccharides
1343	CH_2_ wagging vibrations	Lipids
1306	Amide III	Proteins
1240	Phosphodiester stretch (ν_as_ PO_2_-) from nucleic acids and other phosphorylated molecules	DNA
1211	C–O stretch (νC–O) from free nucleotides	DNA and Polysaccharides
1150	C–O stretch (νC–O) of carbohydrates	Polysaccharides
1117	C–O stretch (νC–O) of carbohydrates	Polysaccharides
1080	Phosphodiester stretch (ν_s_PO_2_-) from nucleic acids and other phosphorylated molecules	DNA and Polysaccharides
1022	β-1,4 glucans	Polysaccharides
993	β-1,6 glucans	Polysaccharides
965	Mannans and the C–O stretch (νC–O) of phosphodiesters and ribose	DNA and Polysaccharides
907	Mannans and DNA, RNA, ribose-phosphate skeletal motions	DNA and Polysaccharides

**Table 2:** Summary of species discriminatory bands as seen in each preparation type from loadings.

Preparation Method	Prominent Bands in PC2 Loadings Plots (cm^−1^)	
Species	*Candida krusei* and *Candida glabrata*	*Candida parapsilosis* and *Candida albicans*
Untreated	1634 Amide I—Proteins 1141-C–O stretch (νC–O) of Carbohydrates—Polysaccharides 989-β-1,6 glucans—Polysaccharides	1166-C–O stretch (νC–O) of Carbohydrates—Polysaccharides 1084-Phosphodiester stretch (ν_s_PO_2_-) from nucleic acids and other phosphorylated molecules—DNA and Polysaccharides 1047-Mannans—Polysaccharides 1010-C–O stretch (νC–O) of Carbohydrates—Polysaccharides 969-Mannans and the C–O stretch (νC–O) of phosphodiesters and ribose—Polysaccharides and DNA
Fixed	1634-Amide I—Proteins 1137-C–O stretch (νC–O) of Carbohydrates—Polysaccharides 985-β-1,6 glucans—Polysaccharides	1162- C–O stretch (νC–O) of Carbohydrates—Polysaccharides 1080-Phosphodiester stretch (ν_s_PO_2_-) from nucleic acids and other phosphorylated molecules—DNA and Polysaccharides 1047-Mannans—Polysaccharides 1010-C–O stretch (νC–O) of Carbohydrates—Polysaccharides
Washed	1145-C–O stretch (νC–O) of Carbohydrates—Polysaccharides 1026-β-1,4 glucans—Polysaccharides 989-β-1,6 glucans—Polysaccharides	1047-Mannans—Polysaccharides 1010-C–O stretch (νC–O) of Carbohydrates—Polysaccharides 969-Mannans and the C–O stretch (νC–O) of phosphodiesters and ribose—Polysaccharides and DNA

**Table 3:** Strain names for *Candida* spp. used.

Species	Isolate Reference
*Candida krusei*	APY47
*Candida parapsilosis*	APY48
*Candida albicans*	APY49
*Candida glabrata*	APY50

## Supplementary Materials

The following are available online, Figure S1: PC1 versus PC2 scores plot of each preparation method and corresponding loadings for PC1, Figure S2: PC1 versus PC2 scores plot for each preparation type and their corresponding PC1 loadings for (A) washed (B) untreated and (C) fixed and Figure S3: K-means cluster analysis for the (A) fixed and (B) washed datasets in the 1400–900 cm^−1^ spectral region.

## Abbreviations

PCA: principal component analysis, KMC: K-means cluster analysis, NAC: non-*albicans Candida,* BC: blood culture, FTIR: Fourier transform infrared spectroscopy, PC: principal components, MVDA: multivariate data analysis, YPD: yeast extract peptone dextrose.
