# Supplementary material for: Influence of the Sample Preparation Method in Discriminating Candida spp. Using ATR-FTIR Spectroscopy
Source: Molecules. 2020 Mar 28;25(7):1551. doi: 10.3390/molecules25071551 (PMC7180699; doi:10.3390/molecules25071551)
Supplement: Supplementary file 1 [file molecules-25-01551-s001.pdf]

molecules

MDPI

Article

Influence of the Sample Preparation Method in Discriminating *Candida Spp.* Using ATR-FTIR Spectroscopy

Savithri Pebotuwa [1,2], Kamila Kochan [2,*], Anton Peleg [3,4], Bayden R. Wood [2] and Philip Heraud [2,5,*]

[1] Department of Microbiology, Monash University, Clayton, Victoria 3800, Australia; savi.pebotuwa@monash.edu
[2] Centre for Biospectroscopy and School of Chemistry, Monash University, Clayton, Victoria 3800, Australia; Bayden.Wood@monash.edu
[3] Infection and Immunity Program, Monash Biomedicine Discovery Institute and Department of Microbiology, Monash University, Clayton, Victoria 3800, Australia; anton.peleg@monash.edu
[4] Department of Infectious Diseases, The Alfred Hospital and Central Clinical School, Monash University, Melbourne, 3004, Victoria 3800, Australia
[5] Department of Microbiology and the Biomedicine Discovery Institute, Monash University, Clayton, Victoria 3800, Australia;
* Correspondence: kamila.kochan@monash.edu (K.K.); phil.heraud@monash.edu (P.H.)

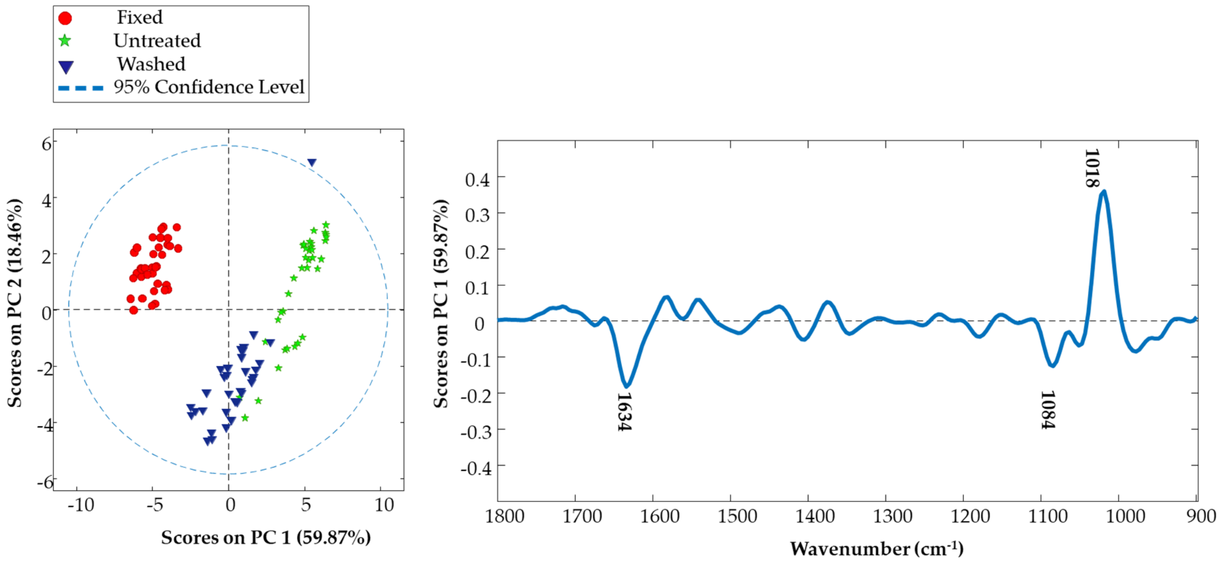

Supplementary materials S1. PC1 versus PC2 scores plot of each preparation method and corresponding loadings for PC1.

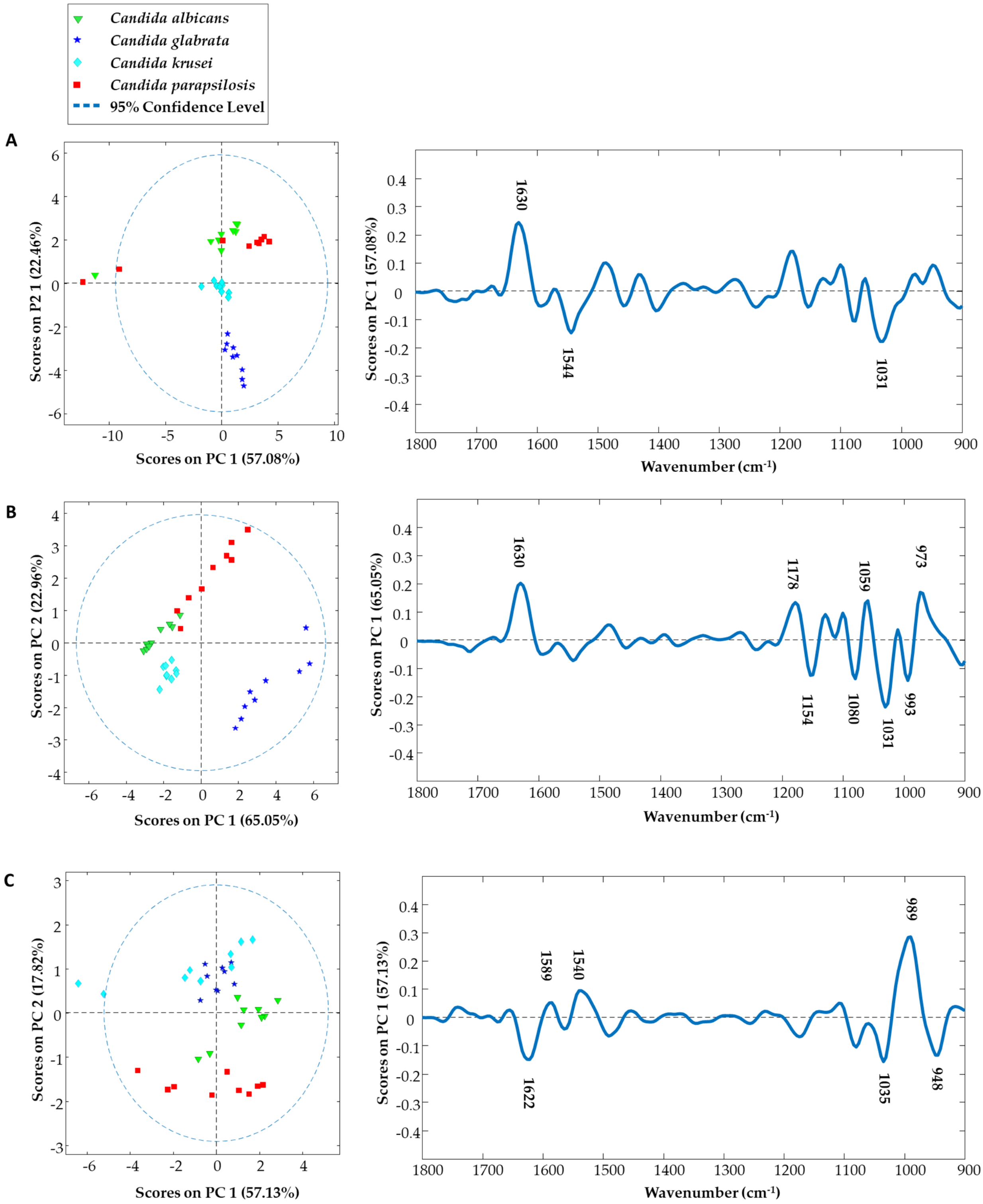

Supplementary materials S2. PC1 versus PC2 scores plot for each preparation type and their corresponding PC1 loadings for (A) Washed (B) Untreated and (C) Fixed.

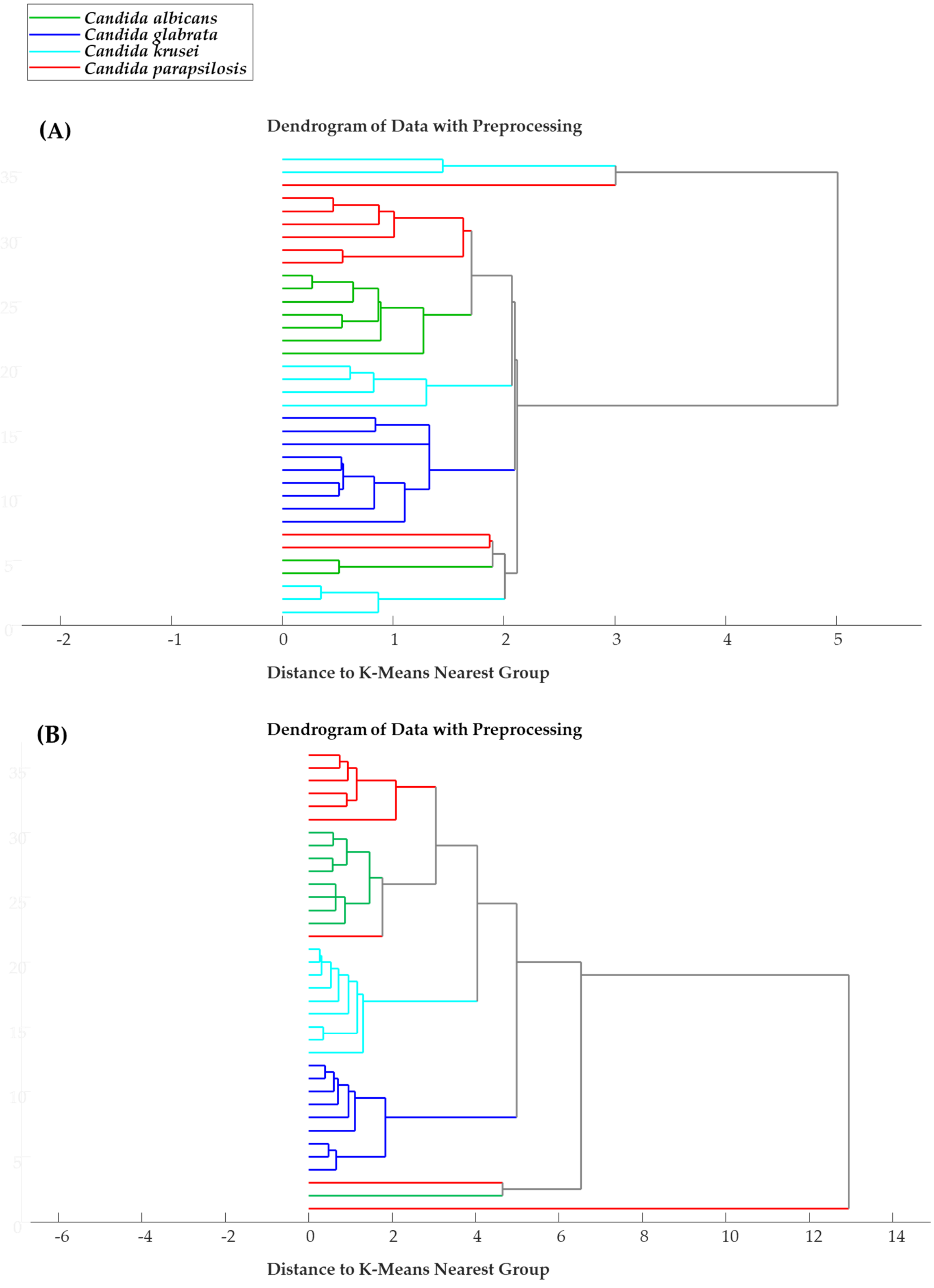

Supplementary materials S3. K-means cluster analysis for the (A) Fixed and (B) Washed datasets in the 1400-900 cm^{-1} spectral region.
